# Supplementary material for: Antagonistic Interaction between Phosphinothricin and Nepeta rtanjensis Essential Oil Affected Ammonium Metabolism and Antioxidant Defense of Arabidopsis Grown In Vitro
Source: Plants (Basel). 2021 Jan 12;10(1):142. doi: 10.3390/plants10010142 (PMC7828019; doi:10.3390/plants10010142)
Supplement: Supplementary file 1 [file plants-10-00142-s001.zip › Supplementary files/Table S1.docx]

**Table S1**: Results of factorial ANOVA on quantities obtained in Arabidopsis shoots and roots, measured 10 days after *in vitro* treatment with BASTA, *N. rtanjensis* essential oil, and of their combinations. The asterisks denote the level of statistical significance: * < 0.05, ** < 0.01 and *** < 0.001. B- BASTA, *Nr*EO - *Nepeta rtanjensis* essential oil.

| Analysis | Figure | Organ | B | *Nr*EO | B+*NrEO* | Box-Cox trans |
| --- | --- | --- | --- | --- | --- | --- |
| Fresh weight | 1C | Shoot | ** |  | * | none |
|  |  | Root | *** |  |  | none |
| Glucose | 1D | Shoot |  |  |  | none |
| Fructose |  |  | ** | ** |  | none |
| Sucrose |  |  | *** | * |  | none |
| Chl a |  |  | *** |  |  | none |
| Chl b |  |  | *** |  |  | none |
| Chl ab |  |  | *** |  |  | none |
| Succinic acid |  |  | *** | *** | ** | none |
| Oxalic acid |  |  | *** |  |  | none |
| Malic acid |  |  | *** | * |  | reciprocal |
| Isocitric acid |  |  | *** | * |  | none |
| Fumaric acid |  |  | * |  |  | none |
| GS activity | 2A | Shoot | *** |  |  | square root |
|  |  | Root | *** | *** |  | square root |
| Ammonia content | 2D | Shoot | *** |  | * | log |
|  |  | Root | *** |  |  | log |
| *GLN1;1* relative expression | 3 | Shoot | *** |  |  | none |
|  |  | Root | *** | * | *** | none |
| *GLN1;2* relative expression |  | Shoot | *** |  |  | none |
|  |  | Root | *** | * | *** | none |
| *GLN1;3* relative expression |  | Shoot | *** |  |  | none |
|  |  | Root | ** | *** | *** | none |
| *GLN1;4* relative expression |  | Shoot | *** |  |  | none |
|  |  | Root |  | * | *** | none |
| *GLN2* relative expression |  | Shoot |  |  |  | none |
|  |  | Root | *** | * | ** | none |
