# Supplementary figures and images for: Antagonistic Interaction between Phosphinothricin and Nepeta rtanjensis Essential Oil Affected Ammonium Metabolism and Antioxidant Defense of Arabidopsis Grown In Vitro
Source: Plants (Basel). 2021 Jan 12;10(1):142. doi: 10.3390/plants10010142 (PMC7828019; doi:10.3390/plants10010142)

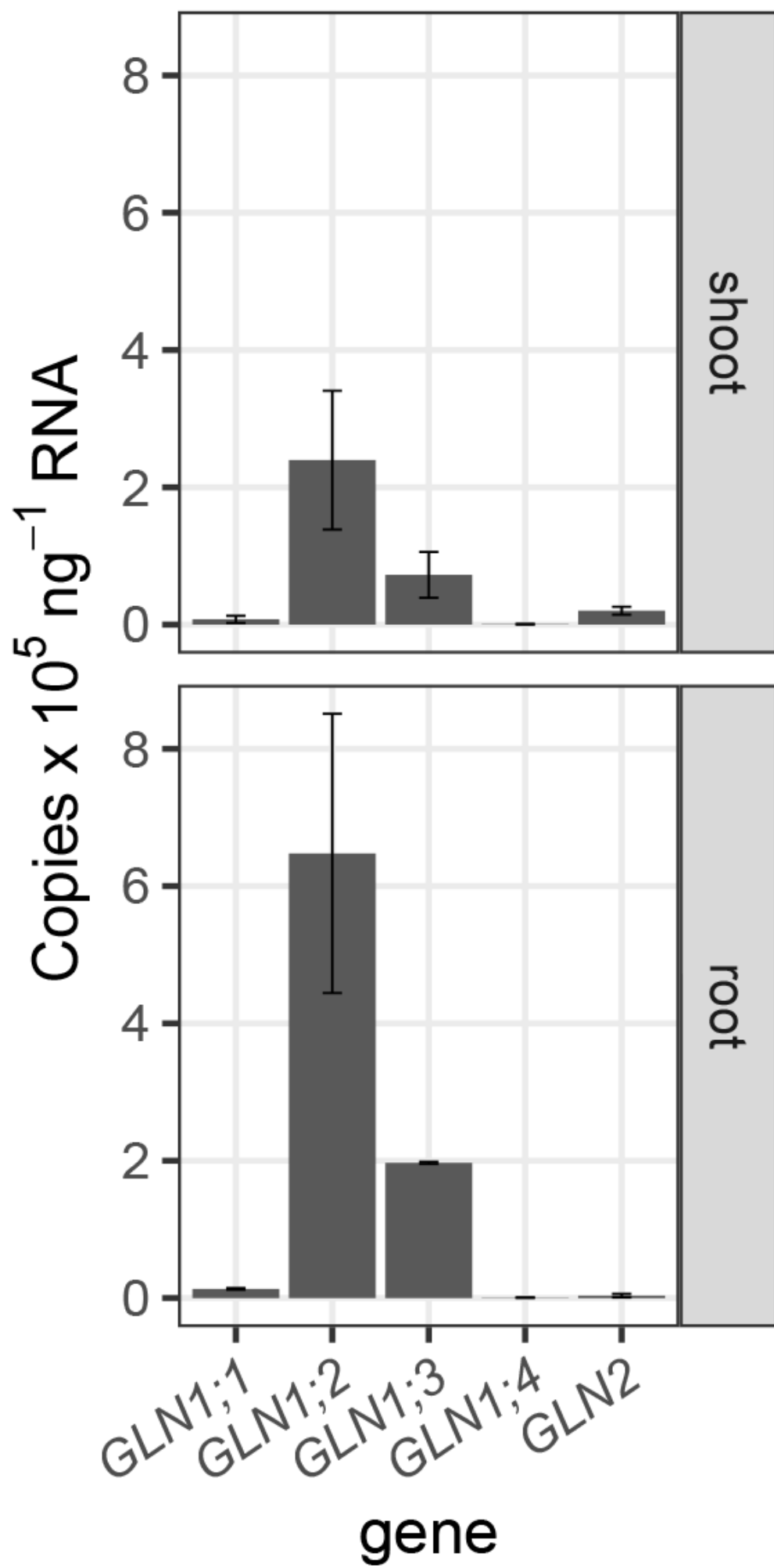

Supplement: Supplementary file 1 [file plants-10-00142-s001.zip › Supplementary files/Figure S1.pdf]
